# Evaluation of Atrial Fibrillation Predictors in ECG After Mitral Valve Repair Surgery in Patients with Mitral Valve Prolapse

**DOI:** 10.3390/medicina61091593

**Published:** 2025-09-04

**Authors:** Aysel Akhundova, Umeyir Savur, Aykun Hakgor, Mehmet Emir Arman, Bilal Boztosun

**Affiliations:** 1 Department of Cardiology, Faculty of Medicine, Istanbul Medipol University, 34214 Istanbul, Turkey; drumeyirsavur@hotmail.com (U.S.); aykunhakgor@gmail.com (A.H.); bboztosun@hotmail.com (B.B.); 2 Internal Medicine, Ascension St. Vincent Hospital-Indianapolis, Indianapolis, IN 46260, USA; dr.mearman@gmail.com

**Keywords:** mitral valve prolapse, mitral valve repair, atrial fibrillation, left atrium, ECG parameters

## Abstract

*Background and Objectives*: Atrial fibrillation (AF) is a common arrhythmia in patients with mitral valve prolapse (MVP) after mitral valve repair surgery and is associated with adverse cardiac outcomes. Early identification of patients at high risk for AF development after repair surgery is crucial for early treatment and follow-up of these patients. This study aimed to identify ECG predictors of AF in patients with MVP following mitral valve repair surgery. *Materials and Methods*: This retrospective, non-randomized study included 62 patients who underwent mitral valve repair for MVP. The patients’ ECGs were analyzed preoperatively and at 1, 3, and 6 months post-surgery to identify patients who developed AF. AF was diagnosed based on ECG findings or Holter monitoring. The P wave dispersion, P wave peak time (PWPT), P wave duration, PR interval, P wave terminal force in lead V1 (PWTF), interatrial block, P wave axis, biphasic P waves in inferior leads, QRS duration, corrected QT interval (QTc), fragmented QRS (fQRS), and ST segment-T wave abnormalities were analyzed on baseline ECG as AF predictors. *Results*: The PWPT, P wave dispersion, and maximum P wave duration were significantly longer on preoperative ECG in patients who developed AF postoperatively compared to those who did not. Biphasic P waves were more frequently observed in patients who developed AF postoperatively. Univariate analysis identified biphasic P waves, P wave dispersion, maximum P wave duration, PWPT, and left ventricular ejection fraction (LVEF) as potential predictors of postoperative AF. However, multivariate analysis revealed that P wave dispersion, PWPT, and left atrial volume index (LAVI) were independent predictors of AF in six months after mitral valve repair. No significant changes were observed in QRS duration, QT interval, or the fQRS. *Conclusions*: The P wave dispersion, PWPT, and LAVI are significant ECG predictors of AF following mitral valve repair surgery in MVP patients. These ECG markers may help identify individuals at higher risk for postoperative AF, allowing for targeted monitoring and management strategies.

## 1. Introduction

Mitral valve prolapse (MVP) is the most common cause of mitral regurgitation (MR), affecting approximately 2–3% of the population [1]. MVP develops as a result of myxomatous degeneration in the valve leaflets or connective tissue disorders. Patients can be asymptomatic or present with palpitation, anxiety, chest pain, syncope, fatigue, or dyspnea. Surgical mitral valve repair (MVR) or mitral valve replacement is the recommended treatment for severe primary MR in MVP patients. Adverse effects of primary MVP include significant MR, heart failure, transient ischemic attacks, cerebrovascular accidents, infective endocarditis, atrial and ventricular arrhythmias, and sudden cardiac death (SCD) [2]. Atrial remodeling and dilation, driven by MR and volume overload, create a substrate for developing atrial arrhythmias, including AF. Numerous studies have shown that AF is common in patients with MR resulting from MVP, with its prevalence being notably higher in those with severe MR [3]. Atrial fibrillation (AF), a common supraventricular arrhythmia, is commonly observed in patients with degenerative MVP and is associated with significant thromboembolic complications. Moreover, the mortality risk associated with stroke in patients with AF is higher compared to strokes in those without AF [4].

According to the European Society of Cardiology (ESC) and the European Association for Cardio-Thoracic Surgery (EACTS) guidelines, the development of AF in patients with severe MR may serve as an early indication for mitral valve surgery [5]. Mitral valve repair is an effective treatment option for patients with severe MR; however, AF remains a common postoperative complication, with an incidence ranging from 37% to 50% following valve surgery [6]. Ngaage et al. showed that preoperative AF has been identified as an important predictor of both surgical risk and postoperative morbidity [7]. Postoperative AF is associated with high mortality rate, stroke, and heart failure rehospitalization [8].

While short-term outcomes after mitral valve repair are well-established, there is limited data regarding the long-term prevention of AF after mitral valve repair surgery. Some studies have demonstrated left ventricular and left atrial reverse remodeling following valve repair surgery, which may influence AF parameters [9].

Left atrium volume index, older age, sternotomy, and heart failure are associated with a high rate of AF after mitral valve surgery. The study showed that age > 58 years, body mass index > 28 kg/m^2^, creatinine clearance < 90 mL/min, reoperative surgery, and preoperative inotropic and intra-aortic balloon pump use were predictors of postoperative AF [10].

Studies have shown that the use of beta-blockers in the preoperative period reduces the risk of AF in the postoperative period [11].

Thyroid disease, especially hyperthyroidism, obesity, and obstructive sleep apnea (OSA), are associated with a high risk of AF incidence [12,13]. Electrical, medical, and spontaneous cardioversion are used in the emergency department to treat newly developed AF. A study investigated patients whose sinus rhythm was restored with spontaneous cardioversion and found that absence of heart failure (HF), a small atrial size, recent-onset AF, rapid atrial fibrillatory rate, and the relationship between a previous AF episode and heart rate/blood pressure were main criteria for determination of spontaneous cardioversion [14].

Identifying high-risk patients for AF helps with early diagnosis and treatment of AF and thromboembolic complications. Atrial predictors, such as PR interval, P wave dispersion, interatrial block, P wave axis, amplitude, and terminal force, along with ventricular predictors like abnormalities in QRS amplitude, morphology, duration, QT interval, ST segment, and T-wave abnormalities, have been associated with increased AF detection on ECG [15].

The objective of this study is to assess the predictors of AF in patients with MVP going to mitral valve repair surgery for early identification of AF.

## 2. Materials and Methods

This retrospective study evaluated 83 patients who underwent mitral valve repair for mitral valve prolapse (MVP) at Medipol University Hospital between January 2018 and December 2023. Sixteen patients were excluded from the study due to atrial fibrillation (AF), and five patients underwent coronary artery bypass grafting (CABG). Patients with preoperative sinus rhythm and no atrial fibrillation (AF) history were included. The patients with a history of preoperative AF, prophylactic maze procedure, rheumatic valve disease, active endocarditis, connective tissue disorders, dilated or hypertrophic cardiomyopathy, congenital heart disease, prior cardiac surgery, or any other moderate-to-severe valve disease were not included. A total of 62 patients were included in the study.

The study complies with the Declaration of Helsinki. The study protocol was approved by the National Medical Ethics Committee (the approval number is 275, and the approval date is 6 March 2025). Additional informed consent was obtained from all individual participants who underwent mitral valve repair surgery. Also, a consent form was obtained from the patients upon hospitalization to allow their data to be used for scientific purposes.

### 2.1. Surgical Technique

Patients underwent valve repair using different techniques depending on their valve pathology (commissuroplasty, quadrangular or triangular resection, valve shift plasty, and neochordae replacement). Most patients underwent complete annuloplasty. Patients with moderate or severe tricuspid regurgitation underwent tricuspid annuloplasty.

During valve repair, myocardial protection was typically achieved through the administration of maintenance cardioplegia retrogradely via a catheter in the coronary sinus. Cold blood cardioplegia was administered at 4 °C, with a blood-to-cardioplegia ratio of 4:1. Doses were administered approximately every 20–25 min. Before the cross-clamp was removed, a dose of warm blood was administered through the retrograde catheter.

### 2.2. Cardiac Function Assessment

Cardiac function was assessed preoperatively and at 1, 3, and 6 months postoperatively using transthoracic echocardiography (TTE) in accordance with the American Society of Echocardiography guideline recommendations [16]. Parameters assessed included left ventricular end-diastolic and end-systolic diameters, left atrial size, left ventricular ejection fraction (EF), systolic pulmonary artery pressure, and left atrial volume index. All echocardiographic evaluations were performed with a GE Philips HealthCare Vivid T8 cardiovascular ultrasound machine, using transthoracic transducers.

### 2.3. Detection of Atrial Fibrillation

Postoperative AF episodes were identified using ECG or ECG-Holter monitoring at 1, 3, and 6 months after surgery during scheduled outpatient cardiology follow-ups. All patients underwent routine follow-up visits with a cardiologist in the outpatient setting post-surgery.

In addition to routine follow-up periods, an ECG was taken during unplanned hospital visits when patients were symptomatic, and the presence of AF was investigated. Preoperative and postoperative 12-lead resting ECGs were recorded, using a filter range of 0.5–150 Hz, with a paper speed of 25 mm/s and a voltage gain of 10 mm/mV. These ECGs were independently analyzed by two cardiologists. An AF episode is defined as an arrhythmia that persists for at least 30 s (or the duration of a 12-lead ECG) on an ECG recording, according to criteria established by the European Heart Rhythm Association (EHRA) guidelines [17].

### 2.4. P-Wave Analysis

The following parameters were assessed on ECG:

- P wave duration was measured manually from the onset to the endpoint of the P wave in lead II.
- P wave dispersion (PWD) is estimated as the difference between maximum and minimum P wave durations in ECG leads. The normal value of PWD is 29 ± 9 ms, and the maximum value is 36 ms. PWD ≥ 40 ms is associated with atrial arrhythmias and atrial fibrillation [18].
- P wave terminal force (PWTF) was calculated by multiplying the amplitude and duration of the terminal negative portion of the P wave in lead V1.
- P wave peak time (PWPT) was measured from the onset to the peak of the P wave in lead Dıı.
- The presence of biphasic (+/−) P waves in the lower leads (II, III, or AVF) was evaluated.
- PR interval was measured from the beginning of the P wave to the beginning of the QRS complex.
- P wave axis values between 0° and 75° were considered normal.
- Interatrial block (IAB) is defined as prolonging the P wave duration ≥ 110 milliseconds.
- The QRS interval length was measured from the beginning of the Q wave to the end of the S wave.

The QT interval was measured from the beginning of the QRS complex to the end of the T wave before returning to baseline when a U wave was present. The corrected QT (QTc) interval was calculated using the Bazett formula (dividing the QT interval by the square root of RR interval [19]).

Data were analyzed to evaluate the correlation between postoperative changes in these parameters and the occurrence of AF.

### 2.5. Statistical Analysis

Statistical analyses were performed using IBM SPSS Statistics version 26 (IBM Corporation, Armonk, NY, USA). The patient cohort was divided into two groups based on the development of postoperative atrial fibrillation (AF). Group comparisons were conducted using a *t*-test for normally distributed continuous variables and a chi-square test for categorical variables. Statistical significance was set at a *p*-value of <0.05. According to the two-tailed independent samples *t*-test analysis using the G-power program (G-power version 3.1, Dusseldorf, Germany) with 95% confidence (1 − α), 49% test power (1 − β) and d = 0.5 effect size, the minimum number of samples required in each group was 31, and the total number of samples was 62. 

As shown in Table 1, the study population was divided into two groups: those with and without postoperative AF. Variables were compared between these two groups. Table 2 presents the results of univariate logistic regression analysis, which was used to identify factors associated with the development of postoperative AF. Based on the univariate analysis and clinical relevance, two separate multivariate logistic regression models were developed to identify independent predictors of postoperative AF. Model 1 included electrocardiographic parameters, while Model 2 incorporated clinical and echocardiographic features. The multivariate analyses identified independent predictors of postoperative AF. The optimal cut-off value for PWPT, PW dispersion, and LAVI values for postoperative AF prediction was revealed by ROC analysis.

## 3. Results

Table 1 compares the preoperative baseline clinical and echocardiographic characteristics, and Table 2 compares the ECG parameters of patients with the development of postoperative atrial fibrillation (AF). The mean age of the study population was 53.4 ± 12.5 years, with 36 male patients (58.1%). Comorbidities included hypertension in 27 patients (43.5%), diabetes mellitus in 8 (12.9%), hyperlipidemia in 10 (16.1%), coronary artery disease in 13 (20%), and obstructive sleep apnea (OSA) in 5 (8.1%), Beta-blocker usage was present in 35 (56.5%), and there was no significant difference between AF-developed and non-developed groups (*p* = 0.937). The mean TSH level was 3 (0.5) μIU/mL, and the mean fT4 was 1.2 (0.1) ng/dL. The mean BMI of the population was 25 (0.9). There was no significant difference in BMI (*p* = 0.671), TSH (*p* = 0.233), fT4 values (*p* = 0.596), and OSA (*p* = 0.763) between the two groups.

The mean left ventricular ejection fraction (LVEF) was 60.1 ± 7.3%. No significant differences in left ventricular end-diastolic diameter (LVEDD) were observed between patients with and without postoperative AF (*p* = 0.590). However, left ventricular end-systolic diameter (LVESD), left atrial (LA) diameter, and left atrial volume index (LAVI) were significantly higher in the AF group (*p* = 0.021; *p* = 0.009; *p* = 0.032). Specifically, LA diameter was larger in patients who developed postoperative AF (49 ± 5.9 mm vs. 45.1 ± 5.4 mm; *p* = 0.009). Systolic pulmonary artery pressure (sPAP) was also significantly higher in the AF group compared to the non-AF group (50 ± 15.2 mmHg vs. 36.7 ± 11.9 mmHg; *p* = 0.001).

There was no significant association between mitral valve prolapse (anterior, posterior, or bileaflet) or tricuspid valve prolapse and the development of postoperative AF.

Maximum P wave duration (101.3 ± 16.5 ms vs. 87.8 ± 15.4 ms; *p* = 0.001), P wave peak time (PWPT) (54.8 ± 9.4 ms vs. 43.2 ± 12.1 ms; *p* = 0.001), and P wave terminal force (PWTF) (32.6 ± 9.2 vs. 27.3 ± 8.1; *p* = 0.001) were significantly longer in the AF group compared to the non-AF group.

Biphasic P waves (19 [90.5%] vs. 20 [48.8%]; *p* = 0.001) and interatrial blocks (6 [28.6%] vs. 3 [7.3%]; *p* = 0.03) were observed more frequently in patients who developed postoperative AF. Furthermore, P wave amplitude was higher in the AF group (2.9 ± 0.48 vs. 2.65 ± 0.59; *p* = 0.05). A significant association was found between increased P wave dispersion and the development of postoperative AF (31.1 ± 14.7 ms vs. 19.1 ± 7.9 ms; *p* < 0.001).

No significant differences were noted in heart rate (*p* = 0.363), PR segment duration (*p* = 0.845), or P wave axis (*p* = 0.698) between the two groups.

In this study, no significant difference was found in the number of patients using beta-blockers in the groups that developed and did not develop AF (*p* = 0.937). There was no significant difference in BMI (*p* = 0.671), TSH (*p* = 0.233), fT4 values (*p* = 0.596), and OSA (*p* = 0.763) between the two groups. The Supplementary Table S1 shows the univariable logistic regression analysis of variables associated of postoperative AF. Table 3 presents the results of the univariable and multivariable logistic regression analysis, which assessed the association of various variables with the development of postoperative AF. The univariate analysis identified several electrocardiographic and clinical parameters as predictors of postoperative AF. These included maximum P wave duration, P wave dispersion, PWPT, PWTF, biphasic P waves, and interatrial blocks, as well as hypertension, coronary artery disease, LVEF, LVESD, LA diameter, LAVI, and sPAP.

The multivariate logistic regression analysis included ECG parameters such as P wave dispersion, PWPT, and PWTF, as well as clinical variables like LAVI, which were found to be independently associated with postoperative AF.

As a result of the multivariate analysis, P wave dispersion, PWPT, and LAVI were identified as significant independent predictors of the development of postoperative AF.

Table 4 shows the ROC analysis of predictors of postoperative AF development. In the ROC analysis, the Pw dispersion cut-off value of 24.5 was determined of postoperative AF with 71.4% sensitivity and 85.4% specificity, the PWPT cut-off value of 48.5 was determined of postoperative AF with 81% sensitivity and 68.3% specificity, and the LAVI cut-off value of 40.8 was determined of postoperative AF with 57.1% sensitivity and 63.4% specificity.

## 4. Discussion

In this study, we aimed to identify electrocardiographic (ECG) parameters that predict the development of atrial fibrillation (AF) following mitral valve repair in patients with mitral valve prolapse (MVP). Our findings indicate that maximum P wave (PW) duration, PW dispersion, PW peak-to-peak time (PWPT), and PW total duration (PWTF) were significantly prolonged in patients who developed AF postoperatively compared to those who remained in sinus rhythm. Additionally, biphasic P waves and intra-atrial blocks were more frequently observed in the AF group. However, multivariate analysis revealed that only PW dispersion, PWPT, and left atrial volume index (LAVI) were independent predictors of postoperative AF, whereas maximum PW duration, PWTF, biphasic P waves, and intra-atrial blocks were not.

Postoperative AF is a common complication after mitral valve surgery, with reported incidences ranging from 20% to 40%. Several factors may contribute to the development of AF, including the patient’s demographic characteristics, surgical technique, and pre-existing comorbidities. Median sternotomy has been identified as an independent risk factor for AF. Additionally, atrial trauma due to surgical manipulation or incision may impair electrical conduction [20]. Paroxysmal AF (PAF) in the early postoperative period may also be triggered by electrolyte imbalances, oxidative stress, inflammation, catecholamine release, and sympathetic–parasympathetic imbalance [21]. Our study focused on ECG changes observed six months postoperatively to determine whether these ECG parameters, which are known to predict AF, are associated with the long-term development of AF. To our knowledge, this is the first study to evaluate AF predictors based on ECG markers specifically in MVP patients undergoing valve repair. Given the known complications of postoperative AF, including stroke, heart failure, prolonged hospitalization, and increased mortality [22], early identification of high-risk individuals is clinically important.

The development of AF after mitral valve surgery is closely related to both age and left atrial size. Although age was not significantly different between groups in our study, patients who developed AF had significantly larger left atrium diameters and LAVI. These findings align with previous studies showing that an enlarged left atrium is a strong predictor of AF in both the early and late postoperative periods [23]. Previous studies have demonstrated that mitral valve repair induces both short- and long-term reverse remodeling of the left atrium (LA), which may influence the development of AF. Factors such as LA and left ventricular (LV) volumes, as well as the severity of mitral regurgitation, play significant roles in influencing long-term LA remodeling. This suggests that reverse remodeling may help prevent AF in the long term after repair surgery. A large LA volume following mitral valve repair has been associated with a higher incidence of AF, stroke, prolonged hospitalization, and increased mortality [24].

In addition to structural changes, ECG markers such as P wave duration and P wave amplitude have also been associated with postoperative AF risk [25]. P wave dispersion (PWD), a simple marker easily measured on an ECG, has been shown to predict AF after cardiac surgery [26].

Although previous studies have demonstrated a significant association between a P wave dispersion (PWD) greater than 40 ms [18,27] and the development of postoperative atrial fibrillation (post-AF), our findings indicate a lower threshold. In the ROC analysis, a cut-off value of 24.5 ms was identified, demonstrating 71.4% sensitivity and 85.4% specificity for predicting post-AF. These results suggest that alterations in the atrial electrical substrate associated with AF may begin at lower PWD values than previously reported.

Our results are consistent with the findings of Doi et al., who reported that longer maximum P wave duration (max PWD) and larger LA volume are associated with increased AF recurrence [28], further supporting the idea that both electrical and structural changes in the heart contribute to the development of AF.

We also observed that patients who developed AF had significantly lower left ventricular ejection fraction (LV EF) and larger left ventricular end-systolic diameter (LVESD). LV dilation and reduced EF contribute to atrial stretch and fibrosis, fostering an arrhythmogenic substrate. Previous studies have shown that the recurrence of AF is less frequent in patients with normal systolic pulmonary artery pressure (SPAP) or reduced postoperative pulmonary pressure [29]. Our study found that SPAP was higher in the AF group compared to those in sinus rhythm.

Although hypertension and CAD were more prevalent in the AF group, their potential confounding effects on the relationship between ECG parameters and postoperative AF highlight the need for larger studies with multivariate adjustments.

This study found an association between certain ECG parameters and the development of postoperative AF. However, further prospective studies are needed to clarify the causal role of these changes and their utility in clinical risk stratification.

## 5. Conclusions

The P wave dispersion, PWPT, and LAVI were significant ECG predictors of AF following mitral valve repair surgery in MVP patients. These markers may help identify individuals at higher risk for postoperative AF, enabling more targeted monitoring and management strategies.

### Limitations

This study has several limitations. First, it was a retrospective analysis conducted on a relatively small cohort of 62 patients with advanced mitral insufficiency and mitral valve prolapse who underwent mitral valve repair. Given that this study was conducted at a single institution with a small cohort, the findings may not be generalizable to broader populations. Multicenter studies with larger patient cohorts are needed to confirm these results.

Due to the method of the study, the exact AF time post-op could not be determined. There may also be recurrent episodes of AF in patients, but this could not be documented due to the method of our study. This may be a limitation of our retrospective study. In addition, survival analysis was not performed in the study population.

The 6-month follow-up period may limit our ability to fully assess the long-term predictive value of these markers. Longer follow-up studies are required for better assessment. Longer-term studies are needed to determine if these markers reliably predict AF in the years following mitral valve repair. Moreover, we observed a higher prevalence of hypertension (HT) and coronary artery disease (CAD) in patients who developed postoperative AF. Since hypertension and coronary artery disease are known risk factors for AF, their potential influence on AF development could not be excluded from this study, and it would be important to control for these variables in future research.

## Figures and Tables

**Table 1:** Comparison of preoperative baseline clinical and echocardiographic characteristics of patients in terms of postoperative atrial fibrillation development.

	Patients Without Postop AF (N = 41, 66.1%)	Patients with Postop AF (N = 21, 33.9%)	Overall Population (N = 62)	*p* Value
Age, years	51.3 (12.9)	57.3 (11.1)	53.4 (12.5)	0.073
Gender, female	14 (34.1%)	12 (57.1%)	26 (41.9%)	0.082
BMI, kg/m^2^	24.9 (1.1)	25 (0.8)	25 (0.9)	0.671
Hypertension	14 (34.1%)	13 (61.9%)	27 (43.5%)	0.037
Hyperlipidemia	7 (17.1%)	3 (14.3%)	10 (16.1%)	0.778
Diabetes Mellitus	3 (7.3%)	5 (23.8%)	8 (12.9%)	0.067
Coronary artery disease	5 (12.2%)	8 (38.1%)	13 (21%)	0.018
OSA	3 (7.3%)	2 (9.5%)	5 (8.1%)	0.763
Beta-blocker usage	23 (56.1%)	12 (57.1%)	35 (56.5%)	0.937
TSH, μIU/mL	2.9 (0.6)	3.1 (0.5)	3 (0.5)	0.233
fT4, ng/dL	1.2 (0.1)	1.2 (0.1)	1.2 (0.1)	0.596
LVEF, %	62.2 (4.4)	55.9 (9.7)	60.1 (7.3)	0.001
LVEDD, mm	57.8 (3.9)	58.5 (4.4)	58 (4.1)	0.590
LVESD, mm	38.8 (3.8)	42.1 (6.2)	39.9 (5)	0.021
LAd, mm	45.1 (5.4)	49 (5.9)	46.4 (5.8)	0.009
LAVi, (mL/m^2^)	37 (7.1)	42.7 (9.5)	39 (8.4)	0.032
sPAP, mmHg	36.7 (11.9)	50 (15.2)	41.2 (14.4)	0.001
AL prolapse	19 (46.3%)	15 (71.4%)	34 (54.8%)	0.060
PL prolapse	36 (87.8%)	18 (85.7)	54 (87.1%)	0.816
Bileaflet prolapse	14 (34.1%)	11 (52.4%)	25 (40.3%)	0.166
TV prolapse	21 (51.2%)	13 (61.9%)	34 (54.8%)	0.424

Abbreviations: AF = atrial fibrillation, BMI= body mass index, OSA = obstructive sleep apnea, TSH = thyroid stimulating hormone, fT4 = free thyroxine, LVEF = left ventricular ejection fraction, LVEDD = left ventricular end-diastolic diameter, LVESD = left ventricular end-systolic diameter, Lad = left atrial diameter, LAVi = left atrial volume index, sPAP = systolic pulmonary artery pressure, AL = anterior leaflet, PL = posterior leaflet, TV = tricuspid valve.

**Table 2:** Comparison of preoperative baseline ECG parameters of patients in terms of postoperative atrial fibrillation development.

	Patients Without Postop AF (N = 41, 66.1%)	Patients with Postop AF (N = 21, 33.9%)	Overall Population (N = 62)	*p* Value
Max PW	87.8 (15.4)	101.3 (16.5)	92.4 (16.9)	0.001
Min PW	68.6 (12.3)	70.1 (12.1)	69.1 (12.1)	0.778
PW dispersion	19.1 (7.9)	31.1 (14.7)	23.2 (12)	<0.001
PWPT	43.2 (12.1)	54.8 (9.4)	47.1 (12.5)	0.001
PWTF	27.3 (8.1)	32.6 (9.2)	29.1 (8.7)	0.051
Biphasic P wave	20 (48.8%)	19 (90.5%)	39 (62.9%)	0.001
P wave axis	38 (92.7%)	20 (95.2%)	58 (93.5%)	0.698
Abnormal axis	7 (17.1%)	3 (14.3%)	10 (16.1%)	0.778
Interatrial block	3 (7.3%)	6 (28.6%)	9 (14.5%)	0.025
P wave amplitude	2.65 (0.59)	2.9 (0.48)	2.75 (0.56)	0.046
PR duration	173.8 (60.8)	156.3 (43.9)	167.8 (55.9)	0.845
Heart rate, bpm	79.7 (13)	85.8 (34.9)	81.7 (22.8)	0.363
QRS duration	90 (1.5)	93.6 (7.7)	91.2 (10.5)	0.568
QTc duration	421 (35.2)	430.8 (19.1)	424.4 (30.9)	0.056
fQRS complex	16 (39%)	6 (28.6%)	22 (35.5%)	0.416

Abbreviations: AF = atrial fibrillation, PW = P wave, PWPT = P wave peak time, PWTF = P wave terminal force, QTc = corrected QT, fQRS = fragmented QRS.

**Table 3:** Multivariable logistic regression analysis investigating independent predictors of the development of postoperative atrial fibrillation.

	Univariate OR (95%CI)	*p*-Value	Multivariate OR (95%CI)	*p*-Value
Model 1				
Max PW	1.052 (1.014–1.091)	0.006	0.938 (0.858–1.024)	0.154
PW dispersion	1.114 (1.040–1.193)	0.002	1.140 (1.035–1.255)	0.008
PWPT	1.093 (1.034–1.155)	0.002	1.132 (1.022–1.255)	0.018
PWTF	1.078 (1.007–1.155)	0.030	0.980 (0.878–1.093)	0.717
Biphasic P wave	9.975 (2.054–48.451)	0.004	2.493 (0.386–16.112)	0.337
Interatrial block	5.067 (1.120–22.919)	0.035	1.376 (0.083–22.859)	0.824
Model 2				
Hypertension	3.134 (1.052–9.339)	0.040	3.307 (0.826–13.231)	0.091
Coronary artery disease	4.431 (1.226–16.012)	0.023	1.351 (0.197–9.290)	0.759
LVEF, %	0.862 (0.772–0.964)	0.009	0.92.2 (0.798–1.066)	0.275
LVESD, mm	1.152 (1.022–1.300)	0.021	1.027 (0.874–1.205)	0.748
LAVi, (mL/m^2^)	1.105 (1.012–1.207)	0.026	1.112 (1.002–1.234)	0.045
sPAP, mmHg	1.078 (1.028–1.131)	0.002	1.050 (0.991–1.112)	0.100

Abbreviations: PW = P wave, PWPT = P wave peak time, PWTF = P wave terminal force, LVEF = left ventricular ejection fraction, LVESD = left ventricular end-systolic diameter, LAVi = Left atrial volume index, sPAP = systolic pulmonary artery pressure.

**Table 4:** ROC analysis of predictors of postoperative AF development.

	Cut-Off Value	Sensitivity	Specifity	AUC	CI 95%	*p* Value
PW dispersion	24.5	71.4	85.4	0.815	0.693–0.938	<0.001
PWPT	48.5	81	68.3	0.757	0.640–0.874	0.001
LAVi, (mL/m^2^)	40.8	57.1	63.4	0.667	0.530–0.804	0.032

Abbreviations: PW = P wave, PWPT = P wave peak time, LAVi = left atrial volume index.

## Data Availability

The data presented in this study are available on request from the corresponding author.

## Supplementary Materials

The following supporting information can be downloaded at: https://www.mdpi.com/article/10.3390/medicina61091593/s1, Table S1: Univariable logistic regression analysis evaluating the association of variables of interest with the development of postoperative atrial fibrillation.

## References

[1] Battaglia V., Santangelo G., Bursi F., Simeoli P., Guazzi M. (2023). Arrhythmogenic Mitral Valve Prolapse and Sudden Cardiac Death: An Update and Current Perspectives. Curr. Probl. Cardiol..

[2] Carbone A., D’Andrea A., Scognamiglio G., Scarafile R., Tocci G., Sperlongano S., Martone F., Radmilovic J., D’Amato M., Liccardo B. (2018). Mitral Prolapse: An Old Mysterious Entity—The Incremental Role of Multimodality Imaging in Sports Eligibility. J. Cardiovasc. Echogr..

[3] Borger M.A., Mansour M.C., Levine R.A. (2019). Atrial Fibrillation and Mitral Valve Prolapse: Time to Intervene?. J. Am. Coll. Cardiol..

[4] Vinding N.E., Kristensen S.L., Rørth R., Butt J.H., Østergaard L., Olesen J.B., Torp-Pedersen C., Gislason G.H., Køber L., Kruuse C. (2022). Ischemic Stroke Severity and Mortality in Patients with and Without Atrial Fibrillation. J. Am. Heart Assoc..

[5] Vahanian A., Beyersdorf F., Praz F., Milojevic M., Baldus S., Bauersachs J., Capodanno D., Conradi L., De Bonis M., De Paulis R. (2022). 2021 ESC/EACTS guidelines for the management of valvular heart disease. Eur. Heart J..

[6] Peretto G., Durante A., Limite L.R., Cianflone D. (2014). Postoperative Arrhythmias after Cardiac Surgery: Incidence, Risk Factors, and Therapeutic Management. Cardiol. Res. Pract..

[7] Ngaage D.L., Schaff H.V., Mullany C.J., Barnes S., Dearani J.A., Daly R.C., Orszulak T.A., Sundt T.M. (2007). Influence of Preoperative Atrial Fibrillation on Late Results of Mitral Repair: Is Concomitant Ablation Justified?. Ann. Thorac. Surg..

[8] Goyal P., Kim M., Krishnan U., Mccullough S.A., Cheung J.W., Kim L.K., Pandey A., Borlaug B.A., Horn E.M., Safford M.M. (2022). Post-operative atrial fibrillation and risk of heart failure hospitalization. Eur. Heart J..

[9] Jansen H.J., Bohne L.J., Gillis A.M., Rose R.A. (2020). Atrial remodeling and atrial fibrillation in acquired forms of cardiovascular disease. Heart Rhythm O2.

[10] Albabtain M.A., Almathami E.A., Alghosoon H., Alsubaie F.F., Abdelaal I.M., Ismail H., Adam A.I., Arafat A.A. (2024). Scores predicting atrial fibrillation after mitral valve surgery: Do we need a more specific score?. J. Arrhythmia.

[11] Sneha K., Saha K.K., Mandal S., Sahoo S.S., Kumar V., Kumar M., Kumari S. (2025). Role of beta-blockers in reducing post-operative atrial fibrillation following cardiac surgery. Bioinformation.

[12] Goldstein S.A., Green J., Huber K., Wojdyla D.M., Lopes R.D., Alexander J.H., Vinereanu D., Wallentin L., Granger C.B., Al-Khati S.M. (2019). Characteristics and outcomes of atrial fibrillation in patients with thyroid disease (from the ARISTOTLE trial). Am. J. Cardiol..

[13] Tavares L., Lador A., Valderrábano M. (2021). Sleep Apnea and Atrial Fibrillation: Role of the Cardiac Autonomic Nervous System. Methodist Debakey Cardiovasc. J..

[14] Mariani M.V., Pierucci N., Piro A., Trivigno S., Chimenti C., Galardo G., Miraldi F., Vizza C.D. (2022). Incidence and Determinants of Spontaneous Cardioversion of Early Onset Symptomatic Atrial Fibrillation. Medicina.

[15] Chousou P.A., Chattopadhyay R., Tsampasian V., Vassiliou V.S., Pugh P.J. (2023). Review Electrocardiographic Predictors of Atrial Fibrillation. Med. Sci..

[16] Mitchell C., Rahko P.S., Blauwet L.A., Canaday B., Finstuen J.A., Foster M.C., Horton K., Ogunyankin K.O., Palma R.A., Velazquez E.J. (2019). Guidelines for performing a comprehensive transthoracic echocardiographic examination in adults: The American Society of Echocardiography recommendations. J. Am. Soc. Echocardiogr..

[17] Tzeis S., Gerstenfeld E.P., Kalman J., Saad E.B., Shamloo A.S., Andrade J.G., Barbhaiya C.R., Baykaner T., Boveda S., Calkins H. (2024). 2024 European Heart Rhythm Association/ Heart Rhythm Society/Asia Pacific Heart Rhythm Society/Latin American Heart Rhythm Society expert consensus statement on catheter and surgical ablation of atrial fibrillation. EP Eur..

[18] Pérez-Riera A.R., de Abreu L.C., Barbosa-Barros R., Grindler J., Fernandes-Cardoso A., Baranchuk A. (2016). P-wave dispersion: An update. Indian Pacing Electrophysiol. J..

[19] Hodges M.S., Salerno D.E.D. (1983). Bazett’s qt correction reviewed: Evidence that a linear qt correction for heart rate is better. J. Am. Coll. Cardiol..

[20] Mihos C.G., Santana O., Lamas G.A., Lamelas J. (2013). Incidence of postoperative atrial fibrillation in patients undergoing minimally invasive versus median sternotomy valve surgery. J. Thorac. Cardiovasc. Surg..

[21] Zakkar M., Ascione R., James A.F., Angelini G.D., Suleiman M.S. (2015). Inflammation, oxidative stress and postoperative atrial fibrillation in cardiac surgery. Pharmacol. Ther..

[22] Lilja M., Leaback R., Banefelt J., Park T.J., Shah D., Ferguson W.G., Friberg Ö. (2024). Postoperative atrial fibrillation is associated with long-term morbidity and mortality in older adults: Analysis from the SWEDEHEART Registry. JTCVS Open.

[23] Ibrahim K.S., Kheirallah K.A., Megdadi M.A. (2022). Enlargement of the Left Atrium Strongly Predicts Postoperative Mortality Following Heart Valve Surgery. Vasc. Health Risk Manag..

[24] Stassen J., van Wijngaarden A.L., Wu H.W., Palmen M., Tomsic A., Delgado V., Bax J.J., Marsan N.A. (2022). Left atrial remodeling after mitral valve repair for primary mitral regurgitation: Evolution over time and prognostic significance. J. Cardiovasc. Dev. Dis..

[25] Kizilirmak F., Demir G.G., Gokdeniz T., Gunes H.M., Cakal B., Guler E., Karaca I.O., Omaygenç M.O., Yılmaz F., Olgun F.E. (2016). Changes in electrocardiographic P wave parameters after cryoballoon ablation and their association with atrial fibrillation recurrence. Ann. Noninvasive Electrocardiol..

[26] Lazzeroni D., Parati G., Bini M. (2016). P-wave dispersion predicts atrial fibrillation following cardiac surgery. Int. J. Cardiol..

[27] Gomaa M.M.M., Elsafty E.E.A., Gomaa H.M.M., Abdulrahim M.M., Eladawy A.H.H. (2024). Study of P wave dispersion in patients with paroxysmal atrial fibrillation and its role in the prediction of atrial fibrillation recurrence. Egypt. Heart J..

[28] Doi A., Takagi M., Katayama H., Yoshiyama T., Hayashi Y., Tatsumi H., Yoshiyama M. (2018). Diagnostic value of electrocardiographic P-wave characteristics in atrial fibrillation recurrence and tachycardia-induced cardiomyopathy after catheter ablation. Heart Vessels.

[29] Zheng T., Zhao Y., Ye Q., Zheng S., Meng F., Hu Q., Zhang H., Han J., Tian B., Zhu J. (2023). Impact of pulmonary arterial systolic pressure on patients with mitral valve disease combined with atrial fibrillation. Front. Cardiovasc. Med..

