# Supplementary material for: Evaluation of Atrial Fibrillation Predictors in ECG After Mitral Valve Repair Surgery in Patients with Mitral Valve Prolapse
Source: Medicina (Kaunas). 2025 Sep 4;61(9):1593. doi: 10.3390/medicina61091593 (PMC12471886; doi:10.3390/medicina61091593)
Supplement: Supplementary file 1 [file medicina-61-01593-s001.zip › medicina-3746419-supplementary.pdf]

Supplementary material Table S1: Univariable logistic regression analysis evaluating the association of variables of interest with the development of postoperative atrial fibrillation.

	**Unadjusted OR**	**95% CI**	**P value**
Age, years	1.041	0.995 – 1.088	0.079
Gender, female	2.571	0.874 – 7.562	0.086
Hypertension	3.134	1.052 – 9.339	0.040
Hiperlipidemia	0.810	0.186 – 3.515	0.778
Diabetes Mellitus	3.958	0.844 – 18.574	0.081
Coronary artery disease	4.431	1.226 – 16.012	0.023
LVEF, %	0.862	0.772 – 0.964	0.009
LVEDD, mm	1.047	0.920 – 1.191	0.486
LVESD, mm	1.152	1.022 – 1.300	0.021
LAd, mm	1.123	1.019 – 1.238	0.019
LAVi, (mL/m^2)	1.105	1.012 – 1.207	0.026
sPAP, mmHg	1.078	1.028 – 1.131	0.002
APL prolapse	2.895	0.937 – 8.946	0.065
PL prolapse	0.833	0.179 – 3.884	0.816
Bileaflet prolapse	2.121	0.726 – 6.200	0.169
TV prolapse	1.548	0.529 – 4.524	0.425
Max PW	1.052	1.014 – 1.091	0.006
Min PW	1.010	0.968 – 1.055	0.642
PW dispersion	1.114	1.040 – 1.193	0.002
PWPT	1.093	1.034 – 1.155	0.002
PWTF	1.078	1.007 – 1.155	0.030
Biphasic P wave	9.975	2.054 – 48.451	0.004
P wave axis	1.579	0.154 – 16.180	0.700
Abnormal axis	0.810	0.186 – 3.515	0.778
Interatrial block	5.067	1.120 – 22.919	0.035
P wave amplitude	2.026	0.773 – 5.307	0.151
PR duration	0.993	0.982 – 1.005	0.254
Heart rate, bpm	1.011	0.988 – 1.035	0.334
QRS duration	1.039	0.979 – 1.103	0.209
QTc duration	1.015	0.989 – 1.042	0.268
fQRS complex	0.625	0.201 – 1.946	0.417
OSA	1.333	0.205 – 8.669	0.763
Beta-blocker usage	1.043	0.361 – 3.0.17	0.937
TSH, µIU/mL	1.691	0.683 – 4.187	0.256
fT4, ng/dL	0.415	0.023 – 7.533	0.552
BMI kg/mì	1.061	0.622-1.812	0.827

Abreveations; , LVEF = left ventricular ejection fraction, LVEDD = left ventricular end-diastolic diameter, LVESD== left ventricular end-systolic diameter, Lad= left atrial diameter, LAVi=Left atrial volume index, sPAP=systolic pulmonary aretry pressure, AL = anterior leaflet, PL= posterior leaflet, TV=tricuspid valve, PW= P wave , PWPT= P wave peak time, PWTF=P wave terminal force , QTc= corrected QT, fQRS = fragmented QRS, OSA= obstructive sleep apnea, TSH= thyroid stimulating hormone, fT4= free Thyroxine BMI= body mass index,
